# *Streptococcus salivarius* subsp. *thermophilus* ST-G30 Prevents Dexamethasone-Induced Muscle Atrophy in C2C12 Myotubes

**DOI:** 10.3390/nu17071141

**Published:** 2025-03-26

**Authors:** Mengjie Li, Seong-Gook Kang, Kunlun Huang, Tao Tong

**Affiliations:** 1 College of Food Science and Nutritional Engineering, China Agricultural University, Beijing 100083, China; mengjieli1112@163.com (M.L.); foodsafety66@cau.edu.cn (K.H.); 2 Key Laboratory of Safety Assessment of Genetically Modified Organism (Food Safety), The Ministry of Agriculture and Rural Affairs of the P.R. China, Beijing 100083, China; 3 Beijing Laboratory for Food Quality and Safety, Beijing 100083, China; 4 Department of Food Engineering and Solar Salt Research Center, Mokpo National University, Muangun 58554, Republic of Korea; sgkang@mokpo.ac.kr

**Keywords:** probiotics, dexamethasone, aging, muscle atrophy, *Streptococcus salivarius* subsp. *thermophilus* ST-G30, *Lacticaseibacillus paracasei* LPc-G110

## Abstract

**Background/Objectives:** Sarcopenia is characterized by loss of muscle mass and strength and is associated with aging. Recently, its links with the gut–muscle axis have been reported, suggesting that probiotics could influence muscle health. **Methods:** In the present study, we investigated the protective roles of two lactic acid bacteria strains, *Streptococcus salivarius* subsp. *thermophilus* ST-G30 (ST-G30) and *Lacticaseibacillus paracasei* LPc-G110 (LPc-G110), on skeletal muscle atrophy induced by dexamethasone (DEX) in C2C12 myotubes. **Results:** Our results demonstrated that ST-G30 significantly alleviated DEX-induced myotube atrophy by increasing the myotubes’ diameter (25.95 ± 1.28 vs. 15.30 ± 0.30 μm, *p* < 0.01), improving the fusion index (48.35 ± 1.75 vs. 22.16 ± 2.36%, *p* < 0.0001), and increasing the protein content (1.78 ± 0.02 vs. 1.56 ± 0.01 mg/mL, *p* < 0.05) and myotube length (0.61 ± 0.05 vs. 0.33 ± 0.01, *p* < 0.05), whereas LPc-G110 showed no significant effect on these phenotypes (*p* > 0.05). Transcriptomic analysis reveals that ST-G30 modulates critical signaling pathways and biological processes related to skeletal muscle health. In the current study, KEGG enrichment analysis and WGCNA enabled identification of the PI3K-Akt signaling pathway as a key regulator of these processes, highlighting its essential role in mitigating DEX-induced muscle atrophy. Furthermore, the overlapping DEGs associated with the PI3K-Akt signaling pathway showed strong correlations with muscle atrophy-related indices. **Conclusions:** These findings underscore the potential of ST-G30 as a promising anti-muscle atrophy supplement and provide valuable insights for developing strategies to prevent and treat glucocorticoid-induced skeletal muscle atrophy.

## 1. Introduction

As the global population transitions into a super-aged society, increasing attention is being paid to geriatric conditions such as sarcopenia, hypertension, and neurodegenerative disorders [1]. Sarcopenia is a form of chronic, progressive muscle atrophy characterized by a decline in muscle mass, strength, and function; it is primarily caused by an imbalance where protein degradation exceeds protein synthesis [2]. Given that skeletal muscle serves as the body’s primary protein reservoir, maintaining muscle health is pivotal for promoting healthy aging, ensuring adequate energy availability, and reducing the risk of metabolic disorders [3]. Decline in muscle mass begins around the age of 40, with sarcopenia being more prevalent in the elderly population, affecting an estimated 5–13% of individuals aged 60–70 years [4,5]. The prevalence of sarcopenia ranges from 9.9% to 40.4% among adults in the community and from 2% to 34% among outpatients, reaching up to 56% in hospitalized patients [6]. Sarcopenia is influenced by various mechanisms, such as loss of alpha motor neurons, overproduction of hormones, oxidative stress, systemic inflammation, and gut microbiota dysbiosis [7,8]. Addressing sarcopenia is critical for improving the quality of life for those affected globally. However, effective pharmacological treatments remain elusive, as no drugs for managing this condition have been approved by the U.S. Food and Drug Administration [9]. Current approaches rely on non-pharmacological interventions, including exercise, physical therapy, and dietary adjustments [10].

Probiotics are living microorganisms that remain active in the intestines when ingested in adequate amounts and provide health benefits to the host [11]. Growing interest has focused on the relationship between gut microbiota and skeletal muscle, commonly known as the gut–muscle axis. Intestinal microbes influence muscle phenotype through mechanisms involving nutrient absorption, energy metabolism, immune function, and insulin sensitivity [12]. Notably, impaired gut function has been linked to skeletal muscle atrophy, implying that the consumption of probiotics may serve as a therapeutic strategy to mitigate sarcopenia.

Lactic acid bacteria (LABs) are among the most widely used microorganisms in probiotic applications [13]. Notably, species such as *Streptococcus salivarius* subsp. *thermophilus* (*S*. *thermophilus*) and *Lacticaseibacillus paracasei* (*L. paracasei*) are recognized for their ability to colonize the intestinal epithelial surface, where they help eliminate harmful microbes and promote host health. Due to their extensive history of safe use, both *S*. *thermophilus* and *L. paracasei* have been granted “Qualified Presumption of Safety” status by the European Food Safety Authority [14,15]. These strains have demonstrated promising potential in supporting gut health [16,17,18], alleviating mental disorders [19], enhancing immune function [20], and improving glycemic control [21]. Furthermore, studies have shown that *S. thermophilus* and *L. paracasei* can enhance muscle mass and functionality in mouse models of aging. Specifically, supplementation with *S. thermophilus* CCFM1095 can modulate the gut microbiota composition in D-galactose-induced aging mice, significantly upregulating the expression of genes related to mitochondrial biogenesis signaling pathways in skeletal muscle [22]. *L. paracasei* PS23 improved muscle aging in D-galactose-induced aging mice by stimulating ghrelin secretion in gastric cells [23]. Additionally, *L. paracasei* P62 alleviated age-related muscle wasting by reducing intestinal inflammation in aged mice, altering the gut microbiota composition, and modulating the gastrocnemius muscle AKT, NF-κB, and/or FOXO3a signaling pathways [24].

Considering the absence of effective interventions, there is an urgent need to identify safe and effective strategies capable of preserving skeletal muscle mass by mitigating muscle degradation without introducing adverse effects. Although some LABs have been reported to alleviate sarcopenia, the roles of *S*. *thermophilus* ST-G30 (ST-G30) and *L. paracasei* LPc-G110 (LPc-G110) in muscle atrophy remain unclear. Therefore, this study investigated the potential of ST-G30 and LPc-G110 to counteract muscle atrophy in dexamethasone (DEX)-induced atrophic C2C12 myotubes. To elucidate the underlying molecular mechanisms, a comprehensive transcriptomic analysis of C2C12 myotubes was conducted, complemented by bioinformatics approaches.

## 2. Materials and Methods

### 2.1. Reagents

DEX (catalog # D4902) was sourced from Sigma-Aldrich (St. Louis, MO, USA). Dulbecco’s modified Eagle’s medium (DMEM, #C11995500BT), 0.25% trypsin–EDTA solution (#25200-056), penicillin–streptomycin solution (#15140-122), horse serum (#16050122), and fetal bovine serum (#10270-106) were acquired from Gibco (Thermo Fisher Scientific, Bohemia, NY, USA). The bicinchoninic acid (BCA) protein assay kit (#P0012) and the Cell Counting Kit-8 (CCK-8, #C0037) were purchased from Beyotime Biotechnology (Shanghai, China).

### 2.2. Study Design

This study employed an in vitro experimental design to evaluate the effects of two strains of lactic acid bacteria, ST-G30 and LPc-G110, on DEX-induced muscle atrophy in C2C12 myotubes. The experimental groups included a control group (no treatment), a DEX group (treated with 50 μM DEX for 48 h), an ST-G30 group (treated with 50 μM DEX and 10^8^ CFU/mL ST-G30 for 48 h), and a LPc-G110 group (treated with 50 μM DEX and 10^8^ CFU/mL LPc-G110 for 48 h). The indexes detected in this study included myotube diameter, fusion index, protein content, and myotube length. Transcriptomic sequencing was used to explore the molecular mechanisms underlying the preventative effects of probiotics on DEX-induced muscle atrophy in vitro.

### 2.3. Preparation of Experimental Samples

ST-G30 and LPc-G110 were provided as lyophilized powder from BioGrowing Co., Ltd. (Shanghai, China). For the in vitro studies, ST-G30 and LPc-G110 were re-suspended in phosphate-buffered saline (PBS, pH 7.2).

### 2.4. Cell Culture and Treatment

C2C12 (#CRL-1772), a mouse myoblast cell line, was purchased from the American Type Culture Collection (ATCC, Manassas, VA, USA). Cells were maintained in DMEM supplemented with 10% fetal bovine serum and 1% antibiotics as previously described [25] and were cultured at 37 °C in a 5% CO_2_ incubator until 90% confluence. Then, the myogenic differentiation medium, consisting of DMEM with 2% horse serum and 1% antibiotics, was used for 4 days to stimulate the differentiation of myoblasts. ST-G30 and LPc-G110 media were prepared in PBS (pH 7.2) and diluted in a differentiation medium to a final 10^8^ CFU/mL concentration. DEX (50 μM) was used for 2 days to induce atrophy in C2C12 cells, with or without probiotic supplementation (10^8^ CFU/mL).

### 2.5. Cell Viability Assay

Cell viability was assessed using the CCK-8 assay. C2C12 cells were plated in 96-well plates and allowed to adhere overnight in growth medium. The cells were subsequently exposed to ST-G30 or LPc-G110 culture media (10^3^, 10^4^, 10^5^, 10^6^, 10^7^, and 10^8^ CFU/mL probiotics in growth medium) and incubated for a further 24 h [26]. The group without probiotics (0 CFU/mL) served as the control, while the blank group consisted of medium alone without cell seeding. Following treatment, the cells in each well were incubated with 10 μL of CCK-8 solution for 3 h. Absorbance at 450 nm was then measured using a microplate reader, and cell viability was calculated using the formula provided:

Cell viability (%) = [OD450_probiotics_ − OD450_blank_]/[OD450_control_ − OD450_blank_] × 100%

### 2.6. Giemsa Staining

To visualize myotubes and nuclei, the cells were stained with Giemsa dye. Differentiating myoblasts were first rinsed with PBS and then fixed in methanol for 10 min, as previously described [27]. After drying for 15 min, Giemsa dye (Solarbio, Beijing, China) was used for 30 min to stain the cells. Subsequently, the myotubes were rinsed 5 times with distilled water. Myotubes and nuclei were visualized and imaged using an Olympus IX71 microscope (Olympus, Center Valley, PA, USA) at 10× magnification.

### 2.7. Determination of Myotube Diameter, Fusion Index, and Myotube Length

Muscle cells with three or more nuclei were identified as myotubes. Myotube size was evaluated by measuring the diameter under each experimental condition, using samples from three independent cultures. The average diameter was determined by calculating the mean of three measurements taken along the length of the myotubes, using Image J software (version 1.53, National Institutes of Health, Bethesda, MD, USA). The fusion index was calculated as the ratio of nuclei within the myotubes to the total number of nuclei. To determine this, at least 1000 nuclei per condition were counted across three independent cultures. Nuclei in individual cells and myotubes were counted using Image J software (National Institutes of Health, Bethesda, MD, USA).

### 2.8. Determination of Total Protein Concentrations

C2C12 myotubes were rinsed three times with ice-cold PBS and then lysed with RIPA buffer (Solarbio, Beijing, China) supplemented with 1 mM phenylmethylsulfonyl fluoride (Solarbio, Beijing, China) on ice. The resulting cell lysates were centrifuged at 14,000× *g* for 5 min at 4 °C, and a bicinchoninic acid (BCA) protein assay kit (Beyotime Biotechnology, Shanghai, China) was used to measure the total protein concentration.

### 2.9. Whole Transcriptome Sequencing

RNA sequencing of C2C12 myotubes was performed using the Majorbio iCloud platform (https://www.majorbio.com/ (accessed on 29 November 2024)). Total RNA was extracted from the cells, and its quality was assessed based on RNA concentration and integrity. An RNA sequencing library was prepared and evaluated for quality before sequencing on the Illumina HiSeq X Ten/NovaSeq 6000 platform. The generated data underwent bioinformatics analysis for comparative group assessment. Differentially expressed genes (DEGs) were identified using DESeq2, with significance set at a fold change (FC) ≥ 2 (or FC ≤ 0.5) and a *p*-value < 0.05. Principal component analysis (PCA) was carried out; volcano plots and Venn diagrams were created to visualize the DEGs, and expression heatmaps were generated using OmicStudio (https://www.omicstudio.cn/tool (accessed on 8 January 2025)) [28].

To investigate the biological functions of the DEGs, KEGG enrichment analysis was conducted via the Majorbio cloud platform. Additionally, weighted gene co-expression network analysis (WGCNA) was performed on genes with a mean transcript per million ≥1 in order to explore gene expression patterns and identify clusters of genes with similar expression profiles.

The STRING database (https://string-db.org (accessed on 8 January 2025)) was used with a minimal interaction score of 0.400 to construct a protein–protein interaction (PPI) network. The DEG list was input into STRING to identify hub genes based on core network connections.

Transcription factor (TF) predictions were generated using the web-based ChEA3 tool (https://amp.pharm.mssm.edu/chea3/ (accessed on 8 January 2025)), integrating ReMap, ENCODE, and other CHIP-seq datasets, along with co-expression data from RNA-seq datasets such as TCGA, GTEx, and ARCHS4. The top 20 TFs were identified based on the DEG list submitted to ChEA3.

### 2.10. Statistical Analysis

All data are presented as mean ± standard error of the mean (SEM). Statistical analysis was performed using one-way ANOVA, followed by Dunnett’s multiple comparisons test, with data processed in GraphPad Prism 9.0 (GraphPad Software, Inc., San Diego, CA, USA). A *p*-value of less than 0.05 was regarded as statistically significant.

## 3. Results

### 3.1. Effect of ST-G30 and LPc-G110 on Cell Viability in C2C12 Cells

**Figure 1:**
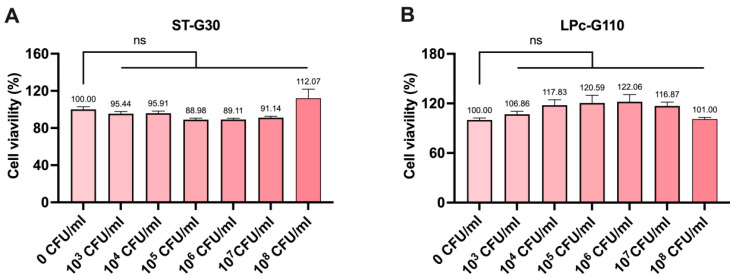
The effects of ST-G30 and LPc-G110 on C2C12 myoblast viability. The cell viability of C2C12 cells was measured after incubation with 0~10^8^ CFU/mL (**A**) ST-G30 or (**B**) LPc-G110 for 24 h. Data are expressed as means ± SEM (*n* = 6). Statistical comparisons between groups are indicated as follows: ns, no significant difference (*p* > 0.05).

The impact of ST-G30 and LPc-G110 on cell viability was assessed using the CCK8 assay. The results demonstrated that treatment with up to 10^8^ CFU/mL of either ST-G30 or LPc-G110 did not have significant cytotoxic effects on the C2C12 myoblasts (Figure 1A,B). Therefore, the concentrations of ST-G30 and LPc-G110 used in the subsequent experiments were both 10^8^ CFU/mL.

### 3.2. Effect of ST-G30 and LPc-G110 on Myotube Formation in DEX-Induced Muscle Atrophy

**Figure 2:**
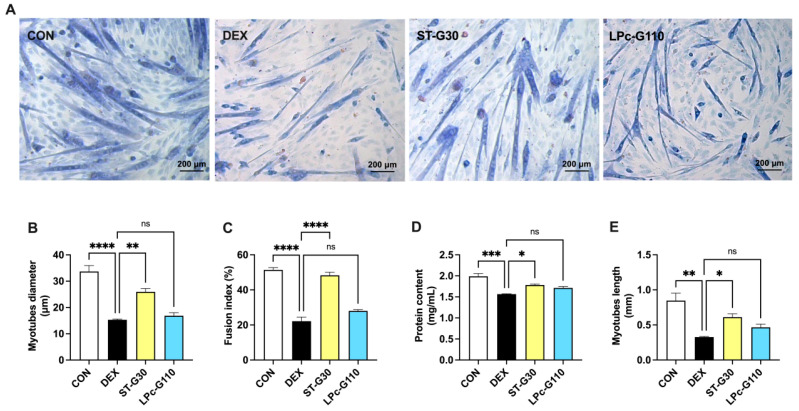
Effects of ST-G30 and LPc-G110 on myotube formation in DEX-induced muscle atrophy. Differentiated C2C12 myotubes were treated with 50 μM DEX and 10^8^ CFU/mL ST-G30 or LPc-G110 for 48 h. (**A**) Representative images of C2C12 myotubes captured using Wright–Giemsa staining under 10× magnification (scale bar = 200 µm). Effects of the probiotics on (**B**) myotube diameter, (**C**) fusion index, (**D**) protein content, and (**E**) myotube length. Data are expressed as means ± SEM (*n* = 3). Statistical comparisons between groups are indicated as follows: * *p* < 0.05; ** *p* < 0.01; *** *p* < 0.001; **** *p* < 0.0001; ns, no significant difference (*p* > 0.05).

To evaluate the effects of ST-G30 and LPc-G110 on the structural integrity of myotubes, Wright–Giemsa staining was performed to estimate changes in myotube diameter, myotube length, and the number of nuclei (Figure 2A). The 50 μM DEX significantly decreased the myotubes’ diameter (Figure 2B), the fusion index (Figure 2C), and the myotubes’ length (Figure 2E). Treatment with ST-G30 significantly attenuated the DEX-induced changes (Figure 2B–E). In addition, the effects of probiotics on total protein content under DEX conditions were determined using a bicinchoninic acid protein assay kit. Remarkably, ST-G30 intervention reversed the DEX-induced decreases in total cellular protein content (Figure 2D). However, treatment with 10^8^ CFU/mL LPc-G110 had no obvious effect on the changes in myotube diameter, fusion index, protein content, and myotube length induced by DEX (Figure 2A–E). These results indicate that ST-G30 can exert a strong preventative effect on DEX-induced myotube atrophy.

### 3.3. Effect of ST-G30 on Transcriptomic Profiles of DEX-Treated C2C12 Myotubes

**Figure 3:**
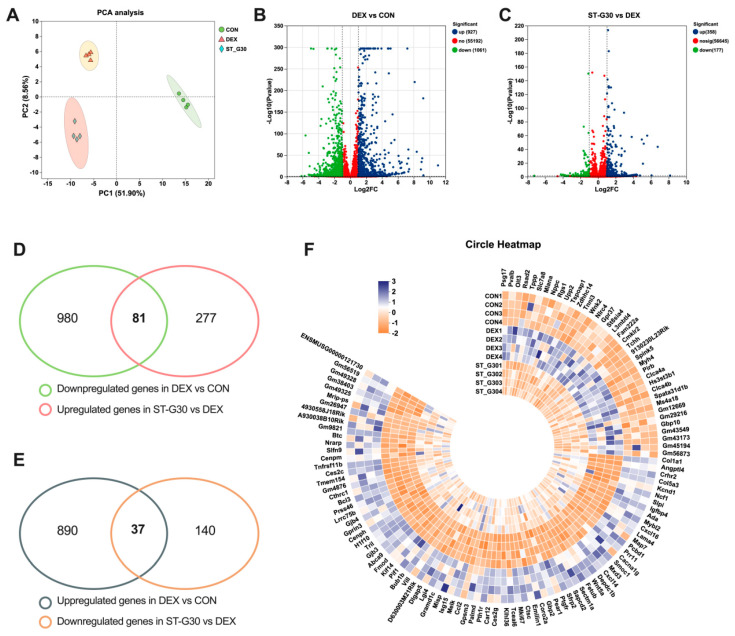
ST-G30 intervention modified the gene expression profiles of DEX-treated C2C12 myotubes. (**A**) PCA of the epididymal WAT transcriptome. (**B**) Volcano plot analysis comparing the DEGs in the DEX group with those in the CON group. (**C**) Similar volcano plot analysis conducted for the DEGs in the ST-G30 group relative to the DEX group. (**D**) Venn diagram analysis revealing 81 genes that were downregulated in the DEX vs. CON comparison and upregulated in the ST-G30 vs. DEX comparison. (**E**) Venn diagram showing 37 genes that were upregulated in the DEX vs. CON comparison and downregulated in the ST-G30 vs. DEX comparison. (**F**) Heatmap of the 118 overlapping DEGs. *n* = 4.

To explore the molecular mechanisms underlying the beneficial effects of ST-G30 on DEX-induced muscle atrophy, RNA sequencing was performed on C2C12 myotubes. PCA revealed a clear separation between the CON, DEX, and ST-G30 groups (Figure 3A). Applying the thresholds of FC ≥ 2 (or FC ≤ 0.5) and *p* < 0.05, a total of 1988 DEGs were identified between the CON and DEX groups. Specifically, 927 genes were significantly upregulated, and 1061 genes were significantly downregulated in the DEX group compared with the CON group (Figure 3B). In the comparison between the ST-G30 and DEX groups, 535 DEGs were identified, with 358 genes showing significant upregulation and 177 genes showing significant downregulation (Figure 3C). Venn diagram analysis showed that 81 overlapping genes were both significantly downregulated by DEX and significantly upregulated by ST-G30, while 37 overlapping genes were significantly upregulated by DEX and significantly downregulated by ST-G30 (Figure 3D,E). Heatmap analysis of these 118 overlapping DEGs demonstrated that ST-G30 effectively reversed the gene expression changes induced by DEX, resulting in a gene expression pattern in the ST-G30 group that more closely resembled that in the control group (Figure 3F). These findings indicate that ST-G30 modulated the gene expression profile in the C2C12 myotubes that had been disrupted by DEX treatment; this may be related to the molecular mechanism by which it alleviates myotubular atrophy.

### 3.4. Pathway Enrichment Analysis Uncovers the Signaling Pathways Regulated by ST-G30

**Figure 4:**
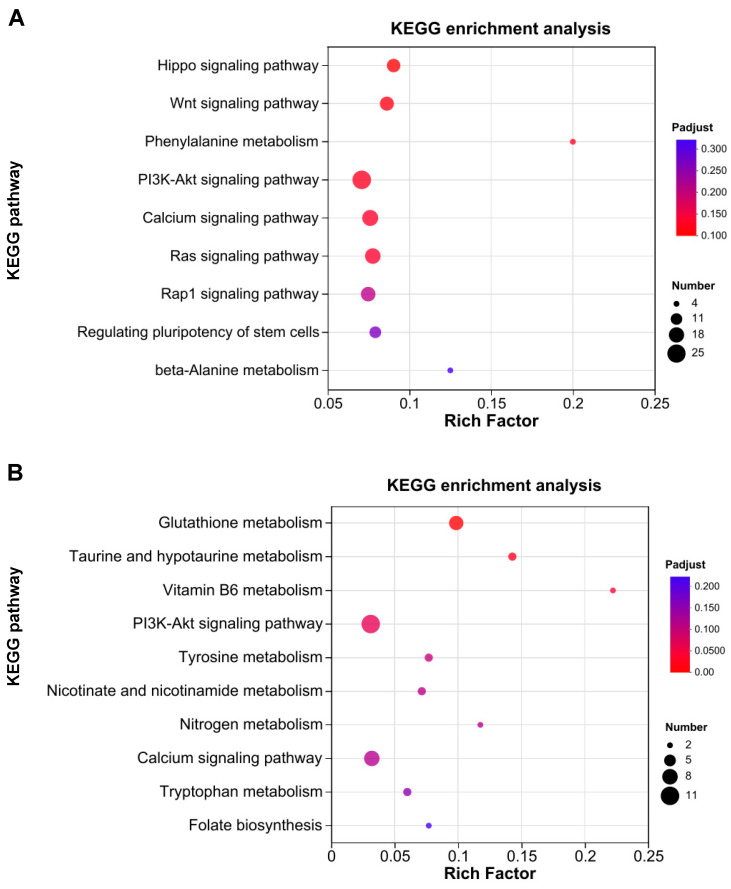
KEGG enrichment analysis of the DEGs in the transcriptome of C2C12 myotubes. (**A**) KEGG pathway enrichment analysis of 1061 DEGs that were significantly downregulated in the DEX group relative to the CON group (*p* < 0.05). (**B**) KEGG pathway enrichment analysis of 358 DEGs that were significantly upregulated in the ST-G30 group relative to the DEX group (*p* < 0.05). *n* = 4.

To further refine the identification of signaling pathways significantly modulated by ST-G30, KEGG enrichment analysis was carried out on the DEGs between the ST-G30 and DEX groups. As illustrated in Figure 4A, a total of 1061 DEGs that were significantly downregulated in the DEX group relative to the CON group were prominently enriched in signaling pathways linked to muscle atrophy, including the calcium signaling pathway, Ras signaling pathway, PI3K-Akt signaling pathway, and Wnt signaling pathway (*p* < 0.05). Figure 4B visualizes the significantly enriched pathways (*p* < 0.05) of the 358 upregulated genes in the ST-G30 group in comparison with the DEX group, highlighting several pathways and biological processes such as glutathione metabolism, taurine and hypotaurine metabolism, vitamin B6 metabolism, the calcium signaling pathway, and the PI3K-Akt signaling pathway. These findings indicate that ST-G30 significantly reversed the inhibitory effect of DEX on genes involved in the PI3K-Akt signaling pathway.

**Figure 5:**
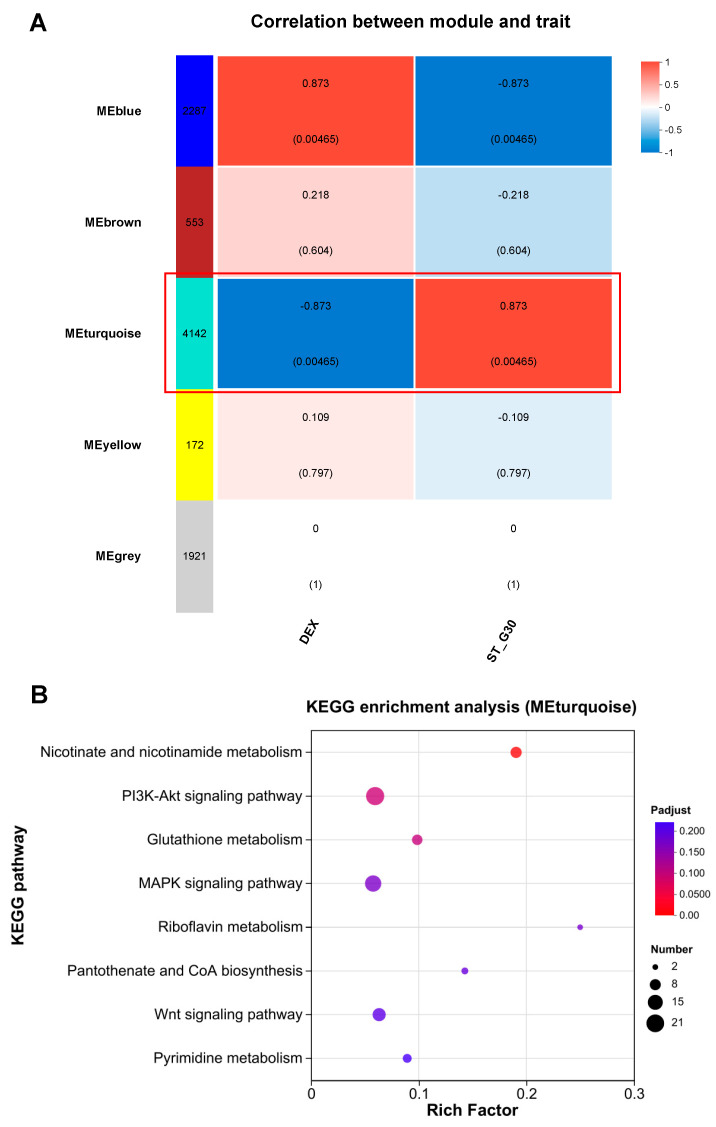
WGCNA of the transcriptome of C2C12 myotubes. (**A**) Heatmap illustrating the module–trait associations, displaying Spearman’s correlation coefficients and corresponding *p*-values between the eigengene values of each module and the treatment groups. (**B**) KEGG enrichment analysis of genes in the MEturquoise module (*p* < 0.05). *n* = 4.

WGCNA is a powerful tool for identifying key gene modules closely associated with specific sample traits [29]. To pinpoint the key gene modules linked to ST-G30 in DEX-treated C2C12 myotubes, WGCNA was conducted using the entire set of genes from the DEX and ST-G30 groups (Figure 5A). Five gene modules were identified, with the MEblue module (2287 genes) and the MEturquoise module (4142 genes) showing significant correlations with the traits of the DEX and ST-G30 groups, respectively, albeit with opposing patterns of association. These two modules were thus designated as characteristic gene modules for the ST-G30-mediated amelioration of myotubular atrophy (Figure 5A). To elucidate the biological mechanisms underlying these modules, KEGG enrichment analysis was performed. The analysis of the MEturquoise module revealed that its 4142 genes were enriched in numerous pathways potentially related to muscle atrophy. Figure 5B highlights all the significantly enriched pathways (*p* < 0.05) that might contribute to the phenotypic changes in muscle atrophy, including nicotinate and nicotinamide metabolism, glutathione metabolism, the Wnt signaling pathway, the MAPK signaling pathway, and the PI3K-Akt signaling pathway.

Thus, the PI3K-Akt signaling pathway was identified as a key signaling pathway associated with muscle atrophy. These results demonstrate that integrating WGCNA and DEG enrichment analyses provided an effective and comprehensive approach to uncover the signaling pathways regulated by ST-G30, suggesting that its protective effects are mediated through modulation of the PI3K-Akt signaling pathway.

**Figure 6:**
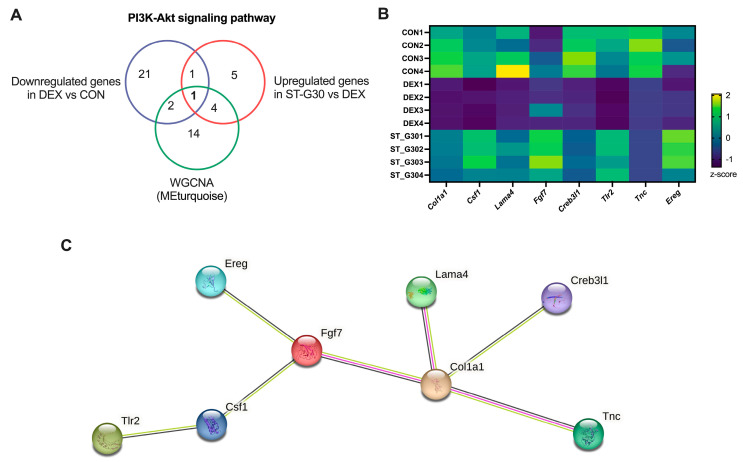
Key genes enriched in PI3K-Akt signaling pathway response to ST-G30 intervention. (**A**) Venn diagrams illustrating overlapping genes enriched in the PI3K-Akt signaling pathway among DEGs downregulated in DEX vs. CON, DEGs upregulated in ST-G30 vs. DEX, and MEturquoise module genes. (**B**) Heatmap of the 8 key genes across the PI3K-Akt signaling pathway, displaying RNA-seq data normalized as z-scores of TPM expression values. (**C**) STRING network visualization of the 8 key genes enriched in the PI3K-Akt signaling pathway. Edges represent interactions between proteins, with the line’s thickness reflecting the strength of the supporting data. *n* = 4.

To identify key genes mediating the protective effects of ST-G30 against DEX-induced muscle atrophy, we analyzed overlapping genes enriched in the PI3K-Akt signaling pathway. Specifically, eight key genes (*Col1a1*, *Csf1*, *Lama4*, *Fgf7*, *Creb3l1*, *Tlr2*, *Tnc*, and *Ereg*) were identified as being enriched in the MEturquoise module, significantly downregulated in the DEX group compared with the CON group, and significantly upregulated in the ST-G30 group compared with the DEX group (Figure 6A,B). To identify key protein-coding gene targets affected by ST-G30 intervention against DEX-induced myotubular atrophy, we performed PPI network analysis using the STRING 12.0 database. The analysis revealed robust correlations among these eight key genes, with *Col1a1* and *Fgf7* identified as the core protein-coding genes in the PPI network (Figure 6C). These findings collectively underscore the potential mediating role of ST-G30 in terms of the mitigating effects on skeletal muscle atrophy that it exerts by modulating critical genes in the PI3K-Akt signaling pathway.

### 3.5. TF Target Enrichment Analysis

**Figure 7:**
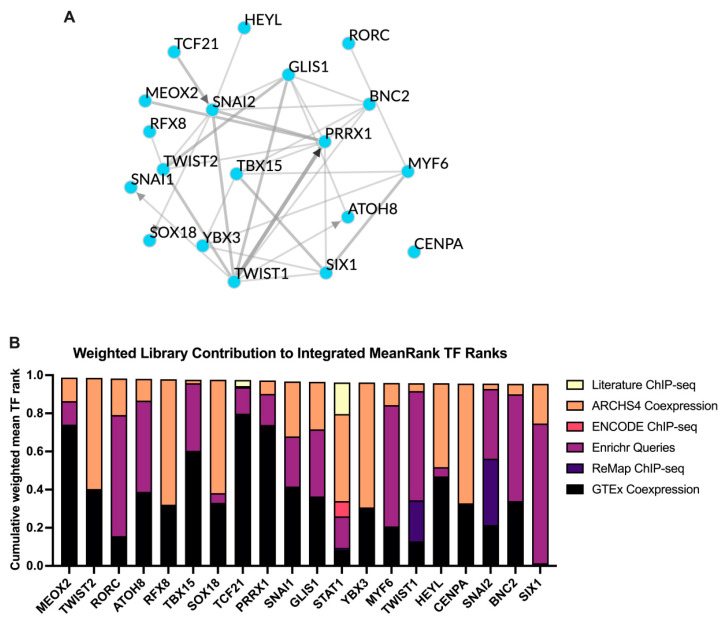
TF analysis of the 358 DEGs upregulated by the ST-G30 group compared with the DEX group. (**A**) The top TF networks generated by ChEA3 using the average integrated ranks across all libraries. Edges between TFs are defined by evidence from ChEA3 libraries, with directional arrows indicating interactions supported by ChIP-seq evidence. (**B**) Bar chart representing the mean ranking of the highest-ranked TFs from ChEA3. The *y*-axis shows the different TFs, while the *x*-axis represents the average mean rank of each TF across all available libraries. *n* = 4.

TF enrichment analysis was conducted using ChEA3 to investigate potential upstream regulators responsible for the observed changes. Based on the mean rank scoring, the top 20 TFs identified were MEOX2, TWIST2, RORC, ATOH8, RFX8, TBX15, SOX18, TCF21, PRRX1, SNAI1, GLIS1, STAT1, YBX3, MYF6, TWIST1, HEYL, CENPA, SNAI2, BNC2, and SIX1 (Figure 7A,B).

### 3.6. Correlation Between the Key DEGs and Muscle Atrophy-Related Indexes

**Figure 8:**
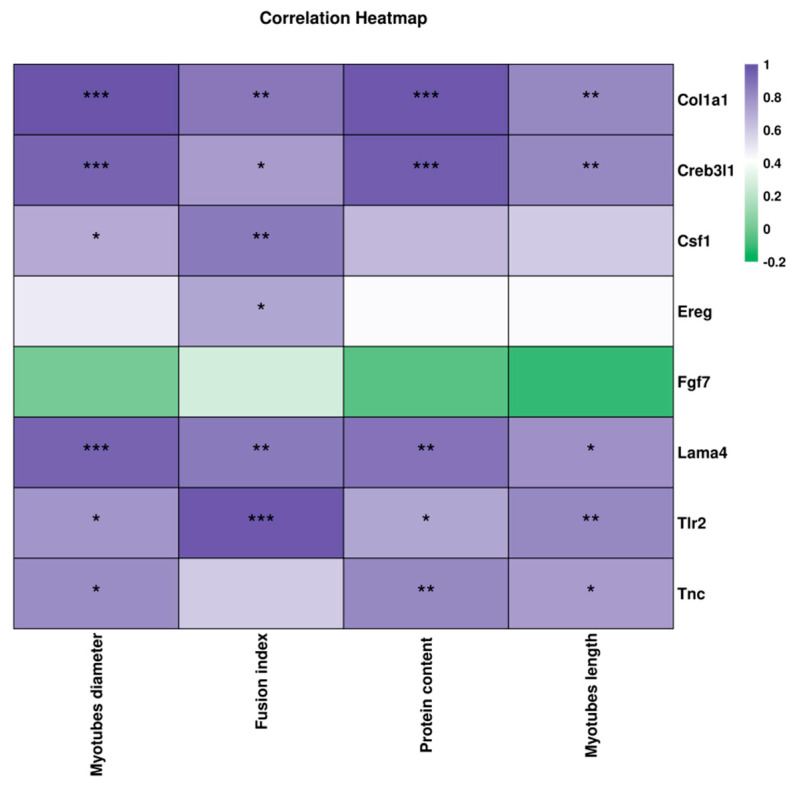
Spearman’s correlation analysis of the 8 key genes enriched in the PI3K-Akt signaling pathway, with muscle atrophy-related indices. Purple shading indicates a positive correlation, green shading indicates a negative correlation, with darker colors representing stronger correlation coefficients. Statistical significance is denoted as * *p* < 0.05, ** *p* < 0.01, and *** *p* < 0.001. *n* = 3.

Correlation analysis revealed a strong positive relationship between the eight identified key genes and indicators relating to skeletal muscle atrophy, highlighting their potential roles in mediating the protective effects of ST-G30 (Figure 8). Specifically, an increase in myotubular diameter was significantly associated with the upregulation of *Col1a1*, *Csf1*, *Lama4*, *Creb3l1*, *Tlr2*, and *Tnc*. Similarly, improvement in the fusion index correlated positively with the upregulation of *Col1a1*, *Csf1*, *Lama4*, *Creb3l1*, *Tlr2*, and *Ereg*. Increases in protein content and the lengths of myotubes were linked to upregulation of *Col1a1*, *Lama4*, *Creb3l1*, *Tlr2*, and *Tnc*. These findings underscore the significant contributions of these genes in counteracting DEX-induced muscle atrophy with ST-G30 intervention.

## 4. Discussion

DEX is a synthetic glucocorticoid that is frequently employed in the treatment of autoimmune diseases such as inflammation, arthritis, and allergies [30]. However, prolonged exposure to high doses of DEX can lead to atrophy of the skeletal muscle. Muscle homeostasis is primarily regulated by the PI3K-AKT-mTOR signaling pathway. DEX inhibits insulin-like growth factor-1 and its binding proteins, leading to suppressed PI3K-AKT signaling and reduced mTOR activity. This cascade diminishes muscle protein synthesis while simultaneously increasing the activity of forkhead box protein O, which accelerates the degradation of muscle protein [31]. Additionally, production of DEX-induced reactive oxygen species exacerbates muscle atrophy by promoting mitochondrial dysfunction and enhancing the ubiquitination of muscle proteins through the upregulation of E3 ligases [32]. Given its role in disrupting muscle homeostasis, DEX has been extensively utilized to induce muscle atrophy both in vitro and in vivo [33,34]. In the current study, treatment with 50 μM DEX significantly reduced the diameter, fusion index, protein content, and length of myotubes compared with the CON group (Figure 2A–E). To investigate potential therapeutic strategies, we assessed the muscle-protective effects of ST-G30 and LPc-G110 in C2C12 myotubes subjected to DEX-induced atrophy.

Probiotics are live microorganisms that confer health benefits to the host when consumed in sufficient quantities. Notably, LABs comprise a key group of probiotics that are increasingly being recognized for their diverse health-promoting effects. *S. thermophilus* is a commensal microorganism naturally found in the human gastrointestinal tract and is commonly used in the production of fermented dairy products [35]. With a long history of safe use, *S. thermophilus* produces bioactive molecules with diverse health-promoting effects. Previous studies have highlighted its benefits, including improved lactose digestion, mitigation of intestinal mucosal inflammation, prevention of colorectal tumor development, modulation of gut microbiota, and regulation of the host metabolism [16,18]. Additionally, the *S. thermophilus* Y2 strain has been shown to produce GABA, a key inhibitory neurotransmitter in the central nervous system, through deep fermentation [36]. The *S. thermophilus* CCFM1312 strain demonstrated the ability to enhance adaptation to activity-based anorexia in mice by modulating gut microbial metabolism and regulating intestinal ghrelin levels [19]. Recent findings further reveal that *S. thermophilus* CCFM1095 alleviates age-related muscle mass loss and functional decline via the gut-muscle axis [22]. Given that *S*. *thermophilus* is one of the most widely consumed bacterial strains in fermented foods, its potential health impacts warrant further investigation.

Our findings confirm that the novel strain ST-G30 at a dose of 10^8^ CFU/mL exhibited significant protective effects against muscle atrophy in DEX-treated C2C12 myotubes (Figure 2A-E). Transcriptome sequencing further revealed that ST-G30 modulated gene expression networks associated with various skeletal muscle processes (Figure 4). Among these, the PI3K-Akt signaling pathway emerged as a key regulatory pathway influenced by ST-G30 supplementation (Figure 6). As previously described, the PI3K-Akt signaling cascade directly regulates protein synthesis, and its inhibition by DEX suppresses this critical process, contributing to muscle atrophy [31]. Our results suggest that ST-G30 may restore PI3K-Akt signaling activity, thereby promoting protein synthesis and alleviating muscle degradation. While these findings provide valuable insights into the molecular mechanisms of ST-G30, further validation through animal models and clinical trials is essential for confirming its efficacy in mitigating skeletal muscle atrophy.

The eight key DEGs enriched in the PI3K-Akt signaling pathway were notably downregulated in the DEX group and upregulated under ST-G30 intervention (Figure 6B, C). As one the fibroblast growth factors, FGF7 promotes myoblast proliferation and delays the myocyte aging process, thereby preventing muscle damage and age-related diseases [37,38]. Additionally, vertical vibration has been shown to upregulate FGF7, facilitating muscle regeneration in C2C12 cells [39]. TnC is a critical regulatory protein for striated muscle contraction, binding to Ca^2+^ and initiating actin–myosin interaction, leading to muscle contraction. Pathogenic variants in the gene encoding fast skeletal TnC can cause mild congenital myopathy, highlighting the importance of TnC in the structure and function of skeletal muscle [40]. Moreover, EREG, an epidermal growth factor regulator, enhances the proliferative capacity of various human cells, including myoblasts, keratinocytes, and fibroblasts. Lin et al. [41] found that tocotrienol-rich fractions promoted the proliferation of aged myoblasts by upregulating EREG expression. In the current study, correlation analysis indicated a robust association between ST-G30’s regulation of *Tnc* and *Ereg* expression and improvements in the values of muscle atrophy markers (Figure 8). Therefore, modulation of these key genes in the PI3K-Akt signaling pathway may underlie ST-G30’s effects in alleviating muscle atrophy, although the precise mechanisms warrant further investigation.

Based on the emerging concept of the gut–muscle axis, the gut microbiota is believed to play a critical role in the development and regulation of skeletal muscle atrophy. Therefore, oral probiotic supplementation has been proposed as a potential strategy for preventing muscle atrophy related to the gut–muscle axis. The mechanisms of action of probiotics are multifaceted, with one key pathway being the modulation of gut microbiota composition [42]. For example, probiotics promote their own growth by competitively excluding pathogenic microorganisms [43]. This regulation of gut microbiota homeostasis facilitates the production of beneficial metabolites, including SCFAs, secondary bile acids, and specific amino acids, which can ultimately modulate muscle function. However, the effects and mechanisms of ST-G30 in modulating gut microbiota require further investigation through animal studies and clinical trials.

Although our study was conducted at the cellular level, the existing literature suggests that similar probiotic interventions have demonstrated promising effects in clinical trials. For instance, *L. paracasei* has been shown to suppress the expression of muscle RING-finger protein-1 and the activation of NF-κB in C2C12 cells [24]. A recent randomized, double-blind clinical trial found that supplementation with live or heat-inactivated *L. paracasei* PS23 for 12 weeks improved lower limb muscle strength and endurance in elderly individuals, although it did not significantly enhance muscle mass. Both forms of supplementation were also shown to effectively reduce inflammatory markers and increase biomarkers associated with muscle synthesis, such as testosterone levels [44]. Additionally, a randomized controlled trial demonstrated that a probiotic composition containing *S. thermophilus* DSM 24,731 improved muscle strength and function in COPD patients by reducing intestinal permeability [45]. Therefore, future animal experiments and clinical trials may validate the beneficial effects of ST-G30 and enable further exploration of its potential application in muscular atrophy-related conditions.

Despite these promising findings, our study has several limitations. Regarding the optimal dosage and treatment duration of probiotics, we selected a maximum non-toxic dose of 10^8^ CFU/mL and a 48 h treatment period based on CCK8 assay results and the previous literature. Future studies may be able to optimize these parameters to determine the most effective dosage and treatment duration. Furthermore, while this study primarily focused on the muscle-protective effects of ST-G30, existing research suggests that probiotics may also impact other organs such as the immune system and gut health. Therefore, future studies could investigate the potential beneficial effects of ST-G30 on other organ functions. Our current results indicate that ST-G30 provides significant protection to muscle function in the short term; however, further research is needed to assess its long-term effects and potential side effects, via animal studies or clinical trials.

## 5. Conclusions

In summary, ST-G30 demonstrated a protective effect against DEX-induced myotubular atrophy by enhancing the myotubes’ diameter, fusion index, protein content, and length. Transcriptomic analysis of C2C12 cells under DEX conditions revealed that ST-G30 significantly influenced multiple signaling pathways and biological processes critical to skeletal muscle health. Notably, the PI3K-Akt signaling pathway was identified as a key regulator in both the KEGG enrichment analysis of DEGs and the WGCNA, underscoring its central role in the protective effects of ST-G30 on DEX-induced muscle atrophy. These findings highlight the potential of ST-G30 as a promising strategic tool for the prevention and treatment of glucocorticoid-induced skeletal muscle atrophy.

## Data Availability

Data supporting reported results can be requested after publication from the corresponding author.

## Abbreviations

The following abbreviations are used in this manuscript:

LABs	Lactic acid bacteria
*S*. *thermophilus*	*Streptococcus salivarius* subsp. *thermophilus*
*L. paracasei*	*Lacticaseibacillus paracasei*
ST-G30	*S*. *thermophilus* ST-G30
LPc-G110	*L. paracasei* LPc-G110
DEX	Dexamethasone
DEGs	Differentially expressed genes
WGCNA	Weighted gene co-expression network analysis
PPI	Protein–protein interaction
TFs	Transcription factor
PCA	Principal component analysis
KEGG	Kyoto Encyclopedia of Genes and Genomes
